# Evaluation of Metabolic Profiles of Patients with Anorexia Nervosa at Inpatient Admission, Short- and Long-Term Weight Regain—Descriptive and Pattern Analysis

**DOI:** 10.3390/metabo11010007

**Published:** 2020-12-24

**Authors:** Manuel Föcker, Alexander Cecil, Cornelia Prehn, Jerzy Adamski, Muriel Albrecht, Frederike Adams, Anke Hinney, Lars Libuda, Judith Bühlmeier, Johannes Hebebrand, Triinu Peters, Jochen Antel

**Affiliations:** 1 Department of Child and Adolescent Psychiatry, University Hospital Münster, Schmeddingstraße 50, 48149 Münster, Germany; 2 Research Unit Molecular Endocrinology and Metabolism, Genome Analysis Center, Helmholtz Zentrum München, German Research Center for Environmental Health, Ingolstädter Landstraße 1, 85764 Neuherberg, Germany; alexander.cecil@helmholtz-muenchen.de (A.C.); prehn@helmholtz-muenchen.de (C.P.); adamski@helmholtz-muenchen.de (J.A.); 3 Department of Biochemistry, Yong Loo Lin School of Medicine, National University of Singapore, 8 Medical Drive, Singapore 117597, Singapore; 4 Chair for Experimental Genetics, Technical University of Munich, 85350 Freising-Weihenstephan, Germany; 5 Department of Child and Adolescent Psychiatry, Psychosomatics and Psychotherapy, University Hospital Essen, University of Duisburg-Essen, Wickenburgstr. 21, 45147 Essen, Germany; murielalbrecht@web.de (M.A.); frederike.adams@googlemail.com (F.A.); Anke.Hinney@uni-due.de (A.H.); Lars.Libuda@uni-due.de (L.L.); Judith.Buehlmeier@uni-due.de (J.B.); Johannes.Hebebrand@uni-due.de (J.H.); Triinu.Peters@uni-due.de (T.P.); Jochen.Antel@uni-due.de (J.A.)

**Keywords:** starvation, metabolomics, anorexia nervosa, metabolites, state marker, trait marker

## Abstract

Acute anorexia nervosa (AN) constitutes an extreme physiological state. We aimed to detect state related metabolic alterations during inpatient admission and upon short- and long-term weight regain. In addition, we tested the hypothesis that metabolite concentrations adapt to those of healthy controls (HC) after long-term weight regain. Thirty-five female adolescents with AN and 25 female HC were recruited. Based on a targeted approach 187 metabolite concentrations were detected at inpatient admission (T_0_), after short-term weight recovery (T_1_; half of target-weight) and close to target weight (T_2_). Pattern hunter and time course analysis were performed. The highest number of significant differences in metabolite concentrations (N = 32) were observed between HC and T_1_. According to the detected main pattern, metabolite concentrations at T_2_ became more similar to those of HC. The course of single metabolite concentrations (e.g., glutamic acid) revealed different metabolic subtypes within the study sample. Patients with AN after short-term weight regain are in a greater “metabolic imbalance” than at starvation. After long-term weight regain, patients reach a metabolite profile similar to HC. Our results might be confounded by different metabolic subtypes of patients with AN.

## 1. Introduction

### 1.1. Anorexia Nervosa

Anorexia nervosa (AN) [1] is a severe, potentially life-threatening mental and nutritional disorder [2] that typically manifests during late childhood and adolescence [3].

Somatic consequences of the starvation associated with AN (but also of other types of starvation) include extreme loss of body fat, a decreased lean body mass, a decreased bone mass, delayed puberty (age of AN onset dependent), amenorrhea, osteoporosis, bradycardia, hypotension and hypothermia. Pseudoatrophy of the brain is a common radiological finding [4]. Clinical laboratory findings are numerous and encompass electrolyte disturbances, hypoglycemia, hypoleptinemia, alterations in micronutrient status and alterations of the hypothalamic-pituitary-gonadal, -thyroid, -adrenal and growth hormone axes [5].

Continuous starvation leading to an emaciated state is distinctly marked by a gross catabolic metabolism [6,7,8]. Relevant central starvation pathways have recently been summarized by Myers and Olson [6]; key signals are insulin and leptin. Leptin levels decrease due to loss of fat mass. Recently, AN was classified as a metabo-psychiatric disorder based on identified genetic correlations with metabolic traits independent of the effects of common variants associated with body-mass index [9].

### 1.2. Metabolomics

Metabolomics attempts to measure most of the small molecule metabolites in a biological system under specific environmental conditions at one specific time point [10]. While metabolomics and metabolites measured in blood or urine might appear distal to brain physiology and mental disorders, the metabolomics footprint might partly (many metabolites are not able to cross the blood brain barrier) mirror metabolic reactions of brain metabolism [11].

Despite these limitations, several recent approaches demonstrate the potential of metabolomics in brain function in general [12,13] but also for specific mental disorders like in schizophrenia [14,15] and major depression [16,17,18].

Metabolomics can contribute to fundamental mechanistic research and the identification of state and potentially trait biomarkers [19] in patients with AN. New biomarkers in this field may lead to the detection of different metabolic subgroups, which require different realimentation strategies concerning weight gain and which imply different prognostic information for the clinician.

A limited number of studies have focused on single or small groups of metabolites, such as tryptophan and other amino acids [20,21,22,23,24], fatty acid compositions [25], lipid metabolism [26,27,28,29,30] and carbohydrates [31,32]. The amino acid tryptophan was shown to be reduced in starved and weight recovered patients with AN [21]. Patients with AN showed deficiencies of selected essential fatty acids and compensatory changes in nonessential fatty acids [25,27,33]. Glucose production was shown to be maintained during starvation [32]. During refeeding, postprandial glucose and 3-O-methylglucose were higher and gastric emptying faster compared with admission [31].

Metabolomics has thus far only rarely been applied to AN. The findings can be summarized as follows: Steroid metabolism was affected both by starvation and realimentation [34]. Epoxide hydroxylase activity was elevated in patients with AN compared to healthy HC [19,34,35,36,37].

A longitudinal association of metabolic factors with the risk of development of eating disorders could be identified [38]. Based on the Avon Longitudinal Study of Parents and Children (ALSPAC) cohort, the relationship of 158 metabolic traits at 7 years (exposure) with the risk for anorexia nervosa at 14, 16 and 18 years was analyzed. Elevated very low-density lipoproteins, triglycerides, apolipoprotein-B/A and monounsaturated fatty acids ratio were inversely associated with AN at age of 18, whereas elevated high-density lipoproteins, docosahexaenoic acid and polyunsaturated fatty acids ratio and fatty acid unsaturation were positively associated with AN at age of 18 years. Nevertheless, specific metabolic traits covering all ages (7, 14, 16, 18 years) could not be clearly identified.

Thus, metabolomic research revealed alterations primarily in steroid and lipid metabolism [19,34,35].

In the only known untargeted fecal metabolomics study of Monteleone and colleagues [39,40] 14 of potentially identified 224 metabolites significantly differentiated underweight patients from weight restored patients and from healthy women.

In our previous work we for the first time applied the targeted metabolomic approach in patients with AN measuring 163 metabolites in 29 adolescent patients with AN in the acute stage of their eating disorder upon inpatient admission (T_0_) and after short-term weight recovery prior to discharge (T_1_; [19]). Because 90 significant metabolite alterations were detected at acute state of starvation compared to HC we concluded that starvation has a substantial impact on metabolite concentrations in patients with AN. Surprisingly, there were larger differences in mean concentrations of metabolites from HC in comparison to those from AN patients after short-term weight recovery as in comparison to those from AN patients at the acute stage of starvation. Thus, the metabolic sequelae of starvation may last longer than expected. The process of realimentation itself may have a stronger impact on metabolite concentrations than a “disease state AN” which persisted already for some time.

Following-up our previous work [19], we now aimed to detect potential state markers via group comparisons of the metabolite concentrations of HC and cases in three weight status dependent measurement time points during the realimentation process (acute starvation at admission for inpatient treatment (T_0_), achievement of half of the target weight (T_1_) and after long term weight regain close to target weight(T_2_) [5]).

We further assessed if the metabolic state in acute AN shifts to a “healthier” metabolite profile (i.e., closer to HC) after long term weight gain. The investigation of metabolite profile changes during the process of refeeding up to a long term weight gain close to target weight (T_2_) with an interim time-point at half of the target weight (T_1_) allows for the assessment of dynamic changes induced via the cessation of starvation and/or the realimentation process.

Finally, we aimed to analyze the homogeneity/heterogeneity of the metabolite dynamics via the time course profiles of AN patients to get information about potentially existing metabolomics based phenotype subgroups (metabotypes).

## 2. Results

The characteristics of the entire study sample and the subsample with complete data for all measurement points are presented in Table 1. Thirty-five female inpatients with AN with a mean age of 15.4 years and a mean duration of illness of 11.5 month were recruited (T_0_), of whom 26 could be followed up at time point T_1_ and 22 at time point T_2_. The mean body mass index (BMI) at referral and at final follow-up were 15.2 kg/m^2^ (mean BMI percentile: 0.98) and 18.5 kg/m^2^ (mean BMI percentile: 16.7), respectively. The characteristics of the subsample with complete data did not differ substantially from the entire study sample. Mean duration of illness in the subsample was 8.8 months versus 11.5 months in the total sample. The mean BMI and mean BMI percentile of HC were 20.7 kg/m² and 39.0 kg/m², respectively. All measured analytes together with concentration means and non-parametric intergroup statistical analyses are displayed in Supplementary Tables S1 and S2.

### 2.1. Group Comparisons

Based on the Kruskal-Wallis test, 46 items, including 40 metabolites, five metabolite ratios and the sum of hexoses were significantly different (Kruskal-Wallis; *p*-value threshold = 2.16 × 10^−4^; Bonferroni correction, Supplementary Table S1) between any time point (T_0_, T_1_, T_2_) and/or HC (Table 2). Pairwise comparisons between HC (analyzed once) and cases at the single time points based on the Mann-Whitney test (Table 2, Supplementary Table S2) revealed few analytes with significant differences at T_0_ (N = 7). We found most analytes significantly different between HC and T_1_ (N = 32) and only little differences between HC and T_2_ (N = 1).

Regarding the course over time, that is, the comparison between cases at each time point based on the Friedman test (Table 2, Supplementary Table S2), most differences in analytes were observed between T_0_ and T_1_ (N = 40). Sixteen and 18 analytes were significantly different regarding the comparisons T_1_ vs. T_2_ and T_0_ vs. T_2_. There was no overlap of significant analytes between the comparisons T_1_ vs. T_2_ and T_0_ vs. T_2_. But nearly all significant analytes of these comparisons were also found to be significant in the comparison between T_0_ and T_1_ (N = 30).

### 2.2. Pattern Hunter

**Figure 1:**
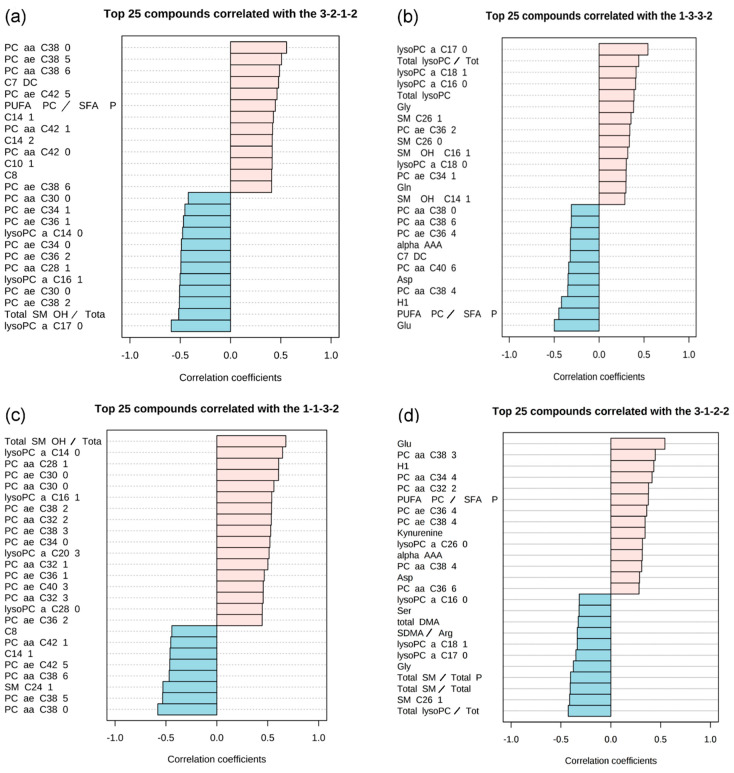
Selected examples of pattern hunter profiles presenting metabolites and their match with the tested pattern. The single images show both positively correlated (in light pink) and negatively correlated (in light blue) metabolites within a vertical bar graph: (**a**) pattern group 3-2-1-2 (HC, T_0_, T_1_,T_2_); (**b**) pattern group 1-3-3-2 (HC, T_0_, T_1_,T_2_); (**c**) pattern group 1-1-3-2 (HC, T_0_, T_1_, T_2_); (**d**) pattern group 3-1-2-2 (HC, T_0_, T_1_, T_2_), 1 < 2 < 3 = relative metabolite concentrations; HC = healthy controls, T_0_ = admission, T_1_ = half of target weight, T_2_ = long-term weight gain close to target weight. Abbreviations see Human Metabolome Database (HMDB) ist: http://www.hmdb.ca/ [42]; correlation coefficient: non-parametric Spearman correlation coefficient of log-transformed measurements.

At first, six main-patterns groups were identified (Table 3). Different profile degrees of steepness (i.e., “2-1-1-2” versus “3-1-1-3”) did not lead to different results. After accounting for the correlation coefficient, there were four different patterns left. The single images show both positively correlated (in light pink) and negatively correlated (in light blue) metabolites within a vertical bar graph (Figure 1).

**Table 3:** Examined patterns of concentrations of metabolites (or their ratios) in healthy controls (HC) and in T_0_, T_1_ and T_2_. Results are grouped according to pattern motives. The most populated individual pattern is ranked highest within each group. The most frequent pattern of all was “1-1-3-2” (=”HC”-“T_0_”-“T_1_”-“T_2_”) which was observed for twelve different analytes.

Pattern Group	Pattern Description	Pattern Tested	N	Best Fitting Analyte (Metabolites, Ratios, Sums) with a Correlation Coefficient > 0.5	r	*p*	Second-Best Fitting Analyte (Metabolites, Ratios, Sums) With a Correlation Coefficient > 0.5	r	*p*
1	The level of metabolites in HC and in T_0_ is comparable. For T_1_ the level rises and is in T_2_ higher or like than in controls.	1-1-3-2	12	Total SM OH/ Total SM non OH	0.68	7.10 × 10^−16^	lysoPC a C14:0	0.65	4.41 × 10^−14^
		2-1-4-3	11	lysoPC a C14:0	0.65	4.37 × 10^−14^	Total SMOH/ Total SM non OH	0.65	4.37 × 10^−14^
		2-1-3-2	10	lysoPC a C14:0	0.63	4.39 × 10^−13^	PC aa C32:2	0.61	3.92 × 10^−12^
2	Metabolite levels in T_1_ (or together with T_2_) have the lowest values. For T_2_ the level is the same as in HC or slightly lower.	2-2-1-1	3	SMC24:1	0.53	3.6 × 10^−9^	PC aa C38:0	0.53	3.90 × 10^−9^
		3-2-1-2	2	PC aa C38:0	0.56	3.73 × 10^−10^	PC ae C38:5	0.51	2.07 × 10^−8^
		2-2-1-2	1	PC aa C38:0	0.53	3.00 × 10^−9^			
		3-2-1-3	0						
3	Metabolite level in T_0_ and T_1_ are higher than in HC. For T_2_ the level approaches that of HC.	1-3-3-2	1	lysoPC a C17:0	0.54	1.22 × 10^−9^			
		1-3-2-1	0						
		1-3-3-1	0						
4	The level of metabolites in T_0_ is much lower than in HC. It rises in T_1_ and T_2._	3-1-2-2	1	Glu	0.54	1.30 × 10^−9^			
		3-1-1-3	0						
		3-1-2-3	0						
5	The level of metabolites is higher in T_0_ than in HC, in T_1_ it is lower or like than in HC. In T_2_ it approaches those of controls	2-3-1-2	0						
		1-2-1-1	0						
6	Theoretically linear increase/decrease assumed. Not observed.	1-2-3-4	1	Total SM OH/ Total SM non OH	0.53	3.63 × 10^−9^			
		4-3-2-1	0			-			

**Figure 2:**
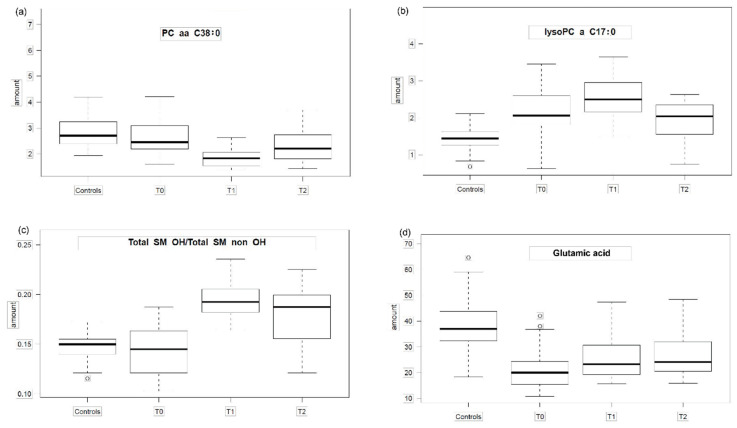
The four main patterns of group means of analyte concentrations. The box plots show concentrations of analytes/metabolites (or metabolite ratios) in HC (healthy controls), T_0_, T_1_ and T_2_ to illustrate 4 different patterns presented in Table 3. (**a**) pattern group 2 (PC aa C38:0: Kruskal-Wallis test *p* = 2.1 × 10^−8^, Table S1); (**b**) pattern group 3 (lysoPC a C17:0 Kruskal-Wallis test *p* = 1.9 × 10^−8^); (**c**) pattern group 1 (total SMOH/total SM non OH Kruskal-Wallis test *p* = 1.0 × 10^−10^); (**d**) pattern group 4 (Glutamic acid Kruskal Wallis test *p* = 3.2 × 10^−7^); T_0_ = admission, T_1_ = half of target weight, T_2_ = long-term weight gain close to target weight. The box and whiskers plot show median (50th percentile) as line, interquartile range (IQR: 25th to the 75th percentile) as box, and minimum and maximum (for minimum and maximum values within Q3 + 1.5*IQR and Q1—1.5*IQR) or Q3 + 1.5*IQR and Q1-1.5*IQR (if minimum and maximum values fell outside Q3 + 1.5*IQR and Q1—1.5*IQR; in that case outlying values are denoted by a circle).

N: Number of analytes (metabolites, ratios, sums) with a correlation coefficient > 0.5; r: Spearman correlation coefficient; *p*: *p*-value for correlation coefficient; lysoPC = (lyso)Phosphatidylcholines, C = Acylcarnitines, H1 = Hexoses, PUFA = Polyunsaturated fatty acids, SM = Sphingomyelines, SFA = Saturated Fatty Acids; Glu = Glutamic acid; HC = healthy controls, T_0_ = admission, T_1_ = half of target weight, T_2_ = long-term weight gain close to target weight.The first pattern observed (Figure 1a and Figure 2a, Supplementary Table S3) shows nearly no difference between HC and T_0_ and a drop of the analyte concentration at T_1_ while the analyte concentration at T_2_ resembles those of HC. The second pattern (Figure 1b and Figure 2b) is characterized by a parabolic shape with a rise of the analyte concentration at T_0_ which rises even further at T_1_ and drops slightly at T_2_ towards the analyte concentration levels observed in HC, however without reaching them. The most prominent pattern describes an analyte concentration profile with no or nearly no difference between HC and T_0_ and a more or less steep rise during realimentation (Figure 1c and Figure 2c) with a subsequent drop of the analyte concentration at T_2_ observed within HC. The fourth pattern (Figure 1d and Figure 2d) comes close to an U-shape with a fall in the analyte concentration levels at T_0_ and a slope upwards at T_1_ and a further upwards trend at T_2_.

### 2.3. Time Course Analysis

**Figure 3:**
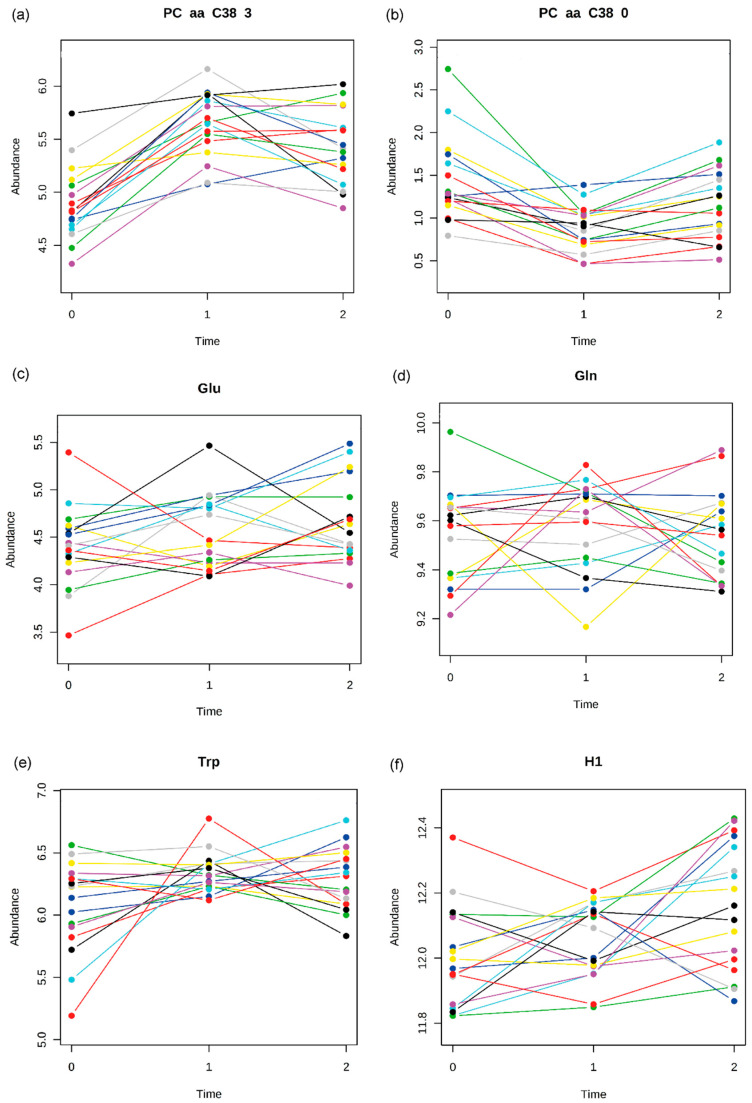
Individual time course profiles of metabolites. Illustration of selected time course profiles (N = 17) of metabolites in T_0_ (0), T_1_ (1) and T_2_ (2) showing either homogenous or heterogeneous patterns. Each line represents the time course profile for one individual patient: (**a**) PC aa C38:3: Phosphatidylcholine (highest Hotelling’s T^2^ in MEBA), (**b**) PC aa C38:0 (best fit in pattern group 2, Table 3), (**c**) Glu: Glutamic acid, (**d**) Gln: Glutamine (**e**) Trp: Tryptophan, (**f**) H1: sum of hexoses (including glucose).

Multivariate Empirical Bayes Analysis (MEBA) was used to analyze time-course profiles in T_0_, T_1_ and in T_2_ for N = 17 patients for whom data for all time points were available (see Supplementary Table S4). The twenty metabolites with the highest Hotellings’s T^2^ values are listed in Table 4. The highest ranked metabolites show the most homogenous albeit also strongest variations over the time course of the analyte/metabolite concentrations as exemplified by PC aa C38:3 and PC aa C38:0 (Figure 3a,b). Whereas analytes further down in the ranking list display a pronounced heterogeneity in their time course profiles as illustrated by the profiles of glutamic acid, glutamine, tryptophan and the sum of hexoses (Figure 3c–f). These analytes show a highly variable pattern consisting of flat courses, U-shapes and inverted U-shapes, pointing either towards a wide variance in general or indicating even metabolically characterizable subgroups of patients, as further elaborated in the discussion section below.

## 3. Discussion

This is to our knowledge the first study with a targeted metabolomics kit for the determination of up to 187 metabolites in patients with AN in comparison to HC.

In the current study, most significant changes in metabolite concentrations were found between acute state of starvation (T_0_) and during weight regain (T_1_) when patients have reached half of their target weight. Contrary to our first study, there were much fewer significant differences between HC and patients at acute state of starvation (T_0_). The pattern analyses confirmed that short term weight regain (T_1_) seems to have a significant impact on the metabolome in comparison to HC and acute state of starvation (T_0_). After long term weight regain close to target weight (T_2_) the analyte concentrations got closer to the healthy state and the metabolome changes/differences appear to decline. Thus, the metabolic state seems to adapt to both the longer-term starvation and the longer lasting weight regain, potentially indicating that a stable metabolic steady state has been reached.

### 3.1. Comparison to the Previous Study

The current study is a consequent sequel of our first study [19] with a substantially modified study design in order to cope with limitations observed within the previous study. The main variation is a different measuring time point T_1_ which was in the first study the time of discharge when most patients had reached their individually defined target weight. In the current study T_1_ denominates the time when patients have reached their half-of-target weight, which means that now the patients are in the peak period of the realimentation process. Since we have realized in the first study that the profiles of metabolites where quite far from control values, that is, out of a balanced metabolism/homeostasis, we introduced in the current study an additional measuring point T_2_ which does now record long term weight regain (close to target weight). Accordingly, a direct comparison of analyte/metabolite concentrations at “old” and “new” T_1_ and/or T_2_ time-points is not meaningful. However, a comparison of the values for HC versus T_0_ out of both studies is justified and should have resulted in similar profiles or profile differences, respectively.

In contrast to our first study which revealed a substantial difference between HC and T_0_ (90 from 112 metabolites showed significantly different serum levels), the current study showed significant deviation for the serum levels of only seven analytes (four metabolites, two ratios and the sum of hexoses).

There are several methodological differences between both studies. Potentially the most important: The blood samples for the first study were taken from multi-centers and were not prepared according to the current strict standard operation procedures (SOPs) for sample preparation but taken from shelved blood samples. This might have led to degradation processes, if immediate sample preparation procedures and strict cooling advices were not exactly followed. The subsequent measurement (including quality checks) of the metabolites followed in both cases the same procedure. However, the applied kit differed only for amino acids, because the p180 is an extension of p150. The differences are: p150 is using only flow injection analysis for all metabolites. p180 is using flow injection analysis for all lipids and hexoses. All amino acids present in p150 and p180 and the additional amino acids and biogenic amines are analyzed in p180 using liquid chromatography. That means: All lipids are quantified using completely the same technology in both assays. It was the Absolute*IDQ*^TM^ p150 Kit (BIOCRATES Life Sciences AG), in the first case whereas we now applied the newer Absolute*IDQ*^TM^ p180 Kit (BIOCRATES Life Sciences AG).

**Figure 4:**
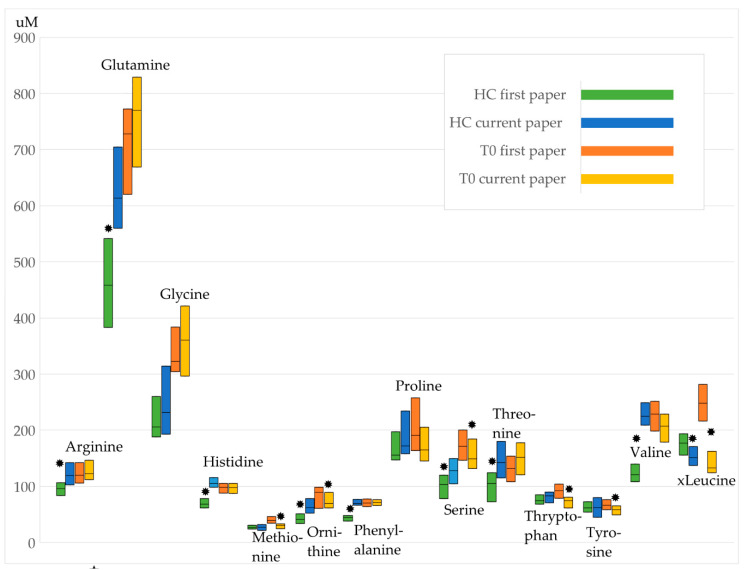
Concentration levels of amino acids as boxplots (25th, 50th, and 75th percentile). Values differ between first and current study: * Mann-Whitney-test *p* < 0.05. The non-normal distribution of concentration levels is illustrated in the Supplementary Figure S1 by violin plots for every amino acid.

Comparing for example the concentration levels of those amino acids which could be analyzed in both studies some significant differences resulted already for the levels found for the healthy controls (see Figure 4, Supplementary Tables S5–S7, Supplementary Figure S1). However, the graphical representation points rather towards a kind of parallel shift of the respective concentration for healthy controls and time point T_0_ indicating that the impact of the disease state on the concentrations is comparable in both studies.

Another reason for the observed divergence might result from the small sample size in bothcases (N = 29 patients [T_0_] first study; N = 35 [T_0_] current study) and the observation of different metabolic endo-phenotypes (see e.g., heterogeneity in time course profiles). This metabolic heterogeneity will be exemplified and discussed in more detail below.

### 3.2. State Markers

The phosphatidylcholine concentrations of PC aa C34:4 and PC aa C38:3 at acute state of starvation (T_0_) were significantly decreased compared to those of HC and patients at T_1_ and T_2_ (Table 2, Supplementary Table S1). The pattern analysis also showed a significant association of these phosphatidylcholine concentrations with the state of starvation (T_0_, see Figure 1d). The time-course patterns of PC aa C34:4 and PC aa C38:3 are characterized by an homogeneous course in regard to test persons and by a strong variation in view of time-points from T_0_ over T_1_ to T_2_ (Table 4). Thus, the two phosphatidylcholines could represent candidates for potential biomarkers with regard to the starvation period. This finding was not supported by our previous study possibly due to the above mentioned methodological explanation.

The concentrations of the phosphatidylcholine PC aa C38:6, the acylcarnitines C10, C10:1, C12:1, C14:1, C14:2 and C7-DC were significantly decreased after short term weight gain (T_1_) compared to those of HC, T_0_ and T_2_ (Table 2). The pattern analysis also revealed a significant association of PC aa C38:6, C10:1, C14:1, C14:2, C7-DC with the state of short term weight gain (T_1_, see Figure 1a and Figure 2a). PC aa C38:6 and C7-DC were characterized by strong and homogenous variations in the respective time courses (Table 4). They seem to reflect metabolic changes after short term realimentation. Whereas, after longer term weight gain, the changes declined. Potentially, theses metabolites could play a role as a screening marker for weight recovery.

A significant increase after short term weight gain (T_1_) could be detected for the lysophosphatidylcholines lysoPC a C14:0, lysoPC a C16:1 and lysoPC a C20:3 (Table 2). The significant association of these metabolites with the state of short term weight gain (T_1_) was confirmed by the pattern analysis (Figure 1c and Figure 2c). LysoPC a C16:1 and lysoPC a C20:3 showed strong and homogeneous variations in the respective time courses (Table 4). Together with PC aa C38:6 and C7-DC, they might become useful as state markers for weight recovery. Since the actually used approach included mainly lipid metabolites the probability to detect changes in the lipid metabolism is obviously increased compared to other biochemical categories (e.g., steroid hormones).

Previous studies have shown that lipid metabolism in patients with AN is altered [25,36]. However, there is a lack of comparability between those studies and ours because of the different metabolomic approaches, measurements and measurement time points. To our knowledge there is no study in which the Absolute*IDQ* Kit p180 was used. In the most recent study of Shih et al. [36], patients with AN showed elevated n-3 PUFAs. Cytochrome P450 pathway oxylipins from arachidonic acid, linoleic acid, alpha-linolenic acid and docosahexaenoic acid PUFAs were associated with AN diagnosis [36]. It was concluded, that PUFA compositions and concentrations may contribute to the pathogenesis and prognosis of AN. Measurements during weight regain were not performed. Based on the measured analytes provided by the kit p180 we were not able to draw conclusions concerning free fatty acids. But we were able to confirm a dysregulation in lipid metabolism both in patients at acute state of starvation and after short term weight recovery.

In the present study glutamic acid was the only amino acid out of 14 whose concentration differed significantly between T_0_/T_1_ and HC. Glutamic acid concentrations were significantly decreased at acute state of starvation followed by a stepwise increase over T_1_ and T_2_. Thus, glutamic acid may represent a state marker for weight recovery. The dysregulated glutamic acid concentration was confirmed by other studies but in the opposite direction [22,23,24]. This discrepancy in group means of metabolite concentrations, such as hyperaminoacidemic and hypoaminoacidemic states, could be due to different metabolic endophenotypes, which is supported by the observation of a very heterogeneous time-course pattern of glutamic acid (Figure 3d). The classification of individuals in subgroups according to their metabolic profile is defined as metabotyping. Based on this approach, one is able to identify differential responses to dietary interventions. Metabotypes might additionally play an important role with regard to starvation and realimentation [43].

Group means of metabolite concentrations at the respective time points (e.g., comparing starved state versus weight recovered state) or in comparison to HC might show up differently from study to study because subgroups with opposite time courses of concentrations profiles—different metabotypes—were possibly included in the study sample randomly. Our time course analysis confirmed considerable variations in the concentration profiles of some metabolites (for example see Figure 3a,b vs. Figure 3c–f). With regard to the glutamic acid and tryptophane concentrations (Figure 3d,e) three subgroups could be identified: (a) a stable course, (b) an U-shaped course and (c) an inversed U-shaped course entailing a lack of significant differences of the mean glutamic acid concentrations between time points (T_0_, T_1_, T_2_) in the total sample. The metabolic “subgroup hypothesis” could also explain the difference to the results of our first study, in which ten amino acids including tryptophane showed a significant difference between HC and T_0_. Overall, a dysregulation of the amino acid pathways in patients with AN seems to be evident. Identification of metabotypes of AN could be the basis for early diagnosis and stratification for targeted therapeutic interventions by metabolic tests as already proposed for patients with autism [44]. The authors identified three autism spectrum disorder related amino acid dysregulation metabotypes.

Metabolomics seems to be a very sensitive tool to detect alterations in the human metabolome prone to a variety of environmental influences, which are not easy to control and to completely consider. Thus, a replication of study results has to be based on “exactly” the same conditions [45].

### 3.3. Limitations

The main limitation is the small sample size and different sized samples at T_0_, T_1_ and T_2_ due to drop outs and early discharge of the patients. Nevertheless, we suspected to detect state markers, because of the enormous effect of starvation and AN on the human metabolism. At a first glance, the results of this study seem to differ substantially from those of our first study [19] (see discussion on amino- acid levels above). However, the impact of the disease state (T_0_) on the concentrations and the direction of concentration changes of analytes seem comparable in both studies if compared to the respective HC. The time-course analysis provide for the first time evidence for the probability of contradicting results for some metabolites in different “small sample size” studies. It has to be considered, that at T_2_ some patients did not reach target weight. After discharge, outpatients were not routinely treated in a standardized manner and did not use professional dietary assessment methods. Thus, metabolite concentrations might also be a result of for example, different dietary components, different nutritional behaviors and a different speed of weight gain. Multiple factors as different energy requirements, different metabolic changes and the stage of treatment contribute to the fact that a uniform weight gain based on a corresponding number of calories cannot be calculated using a mathematical formula [46]. The time to reach half of the target weight and target weight is individually very different and depends besides others on the individual severity and duration of the disorder as well as the adherence of the patients to feeding protocols. This is mirrored by the large variations especially for the time between T_1_ and T_2_. The treatment was not adapted or modified for the study and thus allows the representation of guideline compliant treatment as usual. It is also known that acutely-ill AN patients show increased lipid and lipoprotein concentrations (total cholesterol, High-Density Lipoprotein (HDL), Low-Density Lipoprotein (LDL), triglycerides and apolipoprotein B) as compared to healthy controls [47]. Partially weight-restored AN patients still present with higher total cholesterol and LDL values than healthy controls [47]. In future studies it would be interesting to also measure disease state dependent standard lipid and lipoprotein concentrations in order to compare them with changes in metabolite profiles.

## 4. Materials and Methods

### 4.1. Study Sample

We recruited 35 female adolescents with AN of the restricting type (age range: 12–18 years) at the inpatient unit of the Department of Child and Adolescent Psychiatry, Psychosomatics and Psychotherapy at the University Hospital Essen, Germany and 25 female healthy controls (HC) (age range: 12–18 years). Psychiatric diagnoses were ascertained via clinical examination and the structured interview Diagnostic Interview Assessment-X/ Munich-Composite International Diagnostic Interview (DIA-X/M-CIDI) [48] (according to the Diagnostic and Statistical Manual of Mental Disorders, Fourth Edition [49], Text Revision (DSM-IV-TR). Exclusion criteria for HC and patients with AN were known primary endocrine disorders, intake of any drugs other than psychopharmacological and/or glucocorticoid containing drugs and intellectual disability (i.e., IQ below 70) [50]. HC were recruited from local schools and sports clubs. We excluded psychiatric disorders and severe somatic diseases based mainly on participants’ medical and psychiatric history. The study followed the Declaration of Helsinki and was approved by the ethics committee of the University Hospital Essen, University Duisburg-Essen (12-5289-BO). All participants and their parents provided written informed consent. Finally, 35 female adolescents with AN participate at T_0_, 26 patients were still hospitalized and participated at T_1_ and 22 discharged AN patients could have been followed up for T_2_. Since study participation was voluntary not all initially consenting patients could have been followed-up, because they refused to continue the follow-up assessments. The weight gain experienced during the inpatient treatment was for some patients so aversive and stressful that they could not imagine coming back to the clinic for the T_2_ assessment. Seventeen AN patients were eventually available for measurements at all three time points.

Inpatients with AN were treated according to a multimodal treatment program including weight restoration, medical observation, cognitive–behavioral therapy, family therapy and nutritional counselling. A positive fortifier plan based on body weight gain in 500 g steps was individually established for each patient As suggested in the German guidelines [46], diets contained 30–40 kcal/kg body weight/day at the beginning of treatment. In the further phases of the inpatient weight gain diets were prescribed in such a way that a minimum weight gain of 500 g per week could be maintained. After discharge, outpatients were not routinely treated in a standardized manner.

### 4.2. Anthropometric Assessments

Body weight and height were measured with the same calibrated scales and stadiometers for all participants. Participants were weighed in light underwear without shoes. BMI was subsequently calculated by dividing body-weight by the square of height (kg/m^2^). Individual BMI values were transformed into Standard Deviation Scores (BMI-SDS) with the LMS-Method [51] based on German reference data for children and adolescents [41].

The target weight was individually defined as usual by the clinical stuff according to the German S3-guideline [5].

### 4.3. Sampling, Biochemical Measures and Metabolite Measurement

In general blood sampling was performed at 8 a.m. after an overnight fast. For T_0_ the blood sampling was performed during the first three days of inpatient treatment. For T_1_ and T_2_ blood sampling was performed the day after the achievement of (half of) target weight. Detailed standard operating procedures were followed to safeguard uniform blood sampling. Storing of serum aliquots took place at −80 °C Aliquots were subsequently thawed at room temperature prior to the metabolomics assay (https://www.helmholtz-muenchen.de/fileadmin/GAC/SOPs/20150911__HMGU_Metabolomic_Platform_guidelines.pdf).

We applied the Absolute*IDQ*^TM^ p180 Kit (BIOCRATES Life Sciences AG, Innsbruck, Austria), a commercially available, validated and standardized assay [52] that quantifies up to 188 metabolites in biological samples for targeted metabolomics with 10 mL serum for every time point and study subject. The method of the Absolute*IDQ*^TM^ p180 Kit has been proven to be in conformance with the European Medicines Agency (EMEA)-Guideline “Guideline on bioanalytical method validation (21 July 2011)” [53], which implies proof of reproducibility within a given error range. All analyses were performed in the Helmholtz Zentrum München (GmbH), German Research Center for Environmental Health, Genome Analysis Center.

The detailed sample processing including metabolite description and abbreviations has previously been published [54]. In short, 10 µL of the thawed serum sample were applied directly to the well plate of the p180 kit. Sample handling was performed by a Hamilton Microlab STAR^TM^ robot (Hamilton Bonaduz AG, Bonaduz, Switzerland) and an Ultravap nitrogen evaporator (Porvair Sciences, Leatherhead, UK), beside standard laboratory equipment. Metabolite concentrations were determined by liquid chromatography-electrospray ionization-tandem mass spectrometry (LC-ESI-MS/MS) and flow injection-electrospray ionization-tandem mass spectrometry (FIA-ESI-MS/MS). Mass spectrometric analyses were done on an API 4000 triple quadrupole system (Sciex Deutschland GmbH, Darmstadt, Germany) equipped with a 1200 Series HPLC (Agilent Technologies Deutschland GmbH, Böblingen, Germany) and an HTC PAL autosampler (CTC Analytics, Zwingen, Switzerland) controlled by the software Analyst 1.6.1. Data evaluation for quantification of metabolite concentrations and quality assessment were performed with the Met*IDQ*™ software package, which is an integral part of the Absolute*IDQ*™ Kit. Internal standards were used as a reference for the calculation of metabolite concentrations. The concentrations of the serum samples are given in μmol/L.

The metabolomics dataset enables quantification of up to 188 metabolites out of six physiologically relevant compound classes; that is, 40 acylcarnitines, 21 amino acids, 21 biogenic amines, carbohydrates as “sum of hexoses” (90–95% glucose), 76 phosphatidylcholines, 14 lyso-phosphatidylcholines and 15 sphingomyelins. For the purpose of this study a total of 232 parameters were measured including metabolites, metabolite ratios and sums of metabolites.

### 4.4. Statistical Analysis

We calculated the descriptive statistics (mean, SD, median, minimum and maximum value) of the metabolite concentrations of HC and of the three measurements (T_0_, T_1_ and T_2_) for cases. Duration of illness was calculated as the elapsed time between recalled initiation of weight loss or insufficient age-appropriate weight gain or occurrence of secondary amenorrhea, whatever appeared first and inpatient admission. We compared age, height and body weight (weight, BMI, BMI-SDS) in HC and AN patients at T_0_ using the *T*-test. These analyzes were performed with IBM© SPSS© Statistics (Version 24).

#### 4.4.1. Group Comparisons

First, we compared metabolite concentrations of HC and cases in three measurement time points using non-parametric Kruskal-Wallis test. The test results were corrected for multiple testing using Bonferroni correction for 232 tests (significance threshold *p* = 2.16 × 10^−4^). The changes of analyte concentrations were visualized by boxplots. These analyses were performed on the MetaP publicly available server [55]. Metabolite concentrations between HC and cases at admission (T_0_), at T_1_ and T_2_ were compared with Mann-Whitney-test. The repeated measurements of AN-patients were analyzed using Friedman test with Bonferroni-corrected post-hoc analyses (three tests: T_0_ vs. T_1_, T_0_ vs. T_2_, T_1_ vs. T_2_). We compared amino acid concentrations between HC in the current study and in our first study with Mann-Whitney-test. We performed the same test to compare the concentrations of amino acids in T_0_ between the first and current study. These analyzes were performed with IBM© SPSS© Statistics (Version 24).

#### 4.4.2. Pattern Hunter

In addition to the above mentioned group comparisons, the so-called “PatternHunter” functionality in MetaboAnalyst [56] was applied to enable the identification of metabolites that follow a predefined pattern of concentration changes over the particular sampling points. We applied this module to identify patterns for all metabolites covering the respective serum levels at all time points including HC. The pattern to be tested needs to be specified as a series of numbers separated by “-” [56]. Each number describes the expected relative concentration change at the corresponding sampling point. Thus a pattern designated as “1-2-3-4” would search for metabolites with linearly increasing values across the four corresponding sampling points, whereas a pattern defined as “2-1-1-2” would describe a shallow “U-shape” and the pattern “4-1-1-4” would describe a steeper “U-shape”. We tested in total for all 17 possible permutations with regard to the numbers “1-4” including linear increasing and decreasing, U-shape and inverted U-shape and several Z-shape and inverted Z-shape motives. All theoretically possible linear courses (1-2-3-4 and 4-3-2-1) were tested and documented in Supplementary Table S3. The threshold for the non-parametric Spearman correlation coefficient was set to >0.5. The pattern 1-1-1-1 could not be tested for, since the pattern hunter algorithm cannot deal with a flat, constant concentration profile.

#### 4.4.3. Time Course Analysis

Multivariate Empirical Bayes Analyses (MEBA) was used to analyze time-course profiles in T_0_, T_1_ and in T_2_ (see Supplementary Table S4). In this case, only data from patients with data for all three time-points could be used. MEBA compares time-course mean profiles of each metabolite taking consideration of both within and between time points variance. MEBA ranks metabolites using Hotelling’s T^2^ value. A Hotellings T^2^ test is commonly used when dealing with multi-variate data sets. In these data sets not one variable is assigned to one sample but a multitude of variables. So instead of having to test each variable for all samples separately, Hotellings T^2^ is the analogue of a Student‘s *t*-test for univariate data [57].

The evaluation of this data set by Hotellings T^2^ leads us to the finding, that metabolites with higher Hotelling’s T^2^ value comprise those whose profiles are more different across the time series. To calculate *p*-values for the time-course analysis, we performed the two-way repeated ANOVA. MEBA and ANOVA are integrated in web-based platform MetaboAnalyst [58]. The test results were corrected for multiple testing using Bonferroni correction for 232 tests (significance threshold *p* = 2.16 × 10^−4^).

For pattern hunter analyses and MEBA the metabolite concentrations were normalized using log-transformation.

## 5. Conclusions

PC aa C34:4, PC aa C38:3 and glutamic acid showed-up as potential state markers for the state of acute starvation in AN. PC aa C38:6, C7_DC, lysoPC a C16:1 and lysoPC a C20:3 concentrations seem to reflect the effect of short-term weight regain—a state out of homeostasis—on metabolism. Both, state of starvation and state of weight regain seem to lead to a metabolic homeostasis if they endure for a longer term and become thus more comparable to HC. Further research is needed to replicate these findings and to consolidate the impact of these metabolites as potential state markers. With regard to the future, this research will only be effective, if multicenter metabolomics consortia would provide sufficiently sized patient samples. The final proof for the added value of metabolomics for diagnostic and therapeutic strategies in patients with AN is still missing. In a next step metabolic pathways associated with the above mentioned metabolites will be analyzed and reported in a subsequent publication.

## Figures and Tables

**Table 1:** Characteristics (mean, SD) of the entire study population/* the subset of patients with all measurement points and of healthy controls. Group comparisons T_0_ vs. HC (N = 35, N = 25) were performed with the *T*-test.

Parameter (SD)	T_0_	T_1_	T_2_	HC	T_0_ *	T_1_ *	T_2_ *
Number of patients/HCs	35	26	22	25	17	17	17
Duration of illness (months)	11.47 (8.87)	-	-	-	8.82 (4.05)	-	-
Number of weeks between T_0_ and T_1_		8.05 (2.82)				8.65 (2.24)	
Number of weeks between T_1_ and T_2_ (N = 17)			26.04 (30.58)				26.04 (30.58)
Age (years) ^a^	15.4 (1.4)	15.5 (1.5)	16.0 (1.78)	16.6 (1.8)	15.1 (1.4)	15.3 (1.6)	15.8 (1.8)
Height (cm) ^b^	163.14 (6.60)	163.84 (6.95)	163.85 (6.79)	166.54 (6.00)	163.55 (6.96)	163.64 (7.10)	163.99 (7.20)
Weight (kg) ^c^	40.68 (5.17)	46.01 (5.64)	49.84 (5.83)	57.46 (6.45)	41.32(4.77)	46.19(5.02)	49.59 (6.22)
BMI (kg/m^2^) ^d^	15.24 (1.17)	17.05 (1.15)	18.51 (1.20)	20.70 (1.86)	15.41 (1.03)	17.16 (1.03)	18.38 1.28)
BMI percentile ^+^	0.98 (1.59)	6.86 (6.50)	16.69 (11.09)	39.03 (22.10)	1.30 (1.96)	7.66 (6.91)	16.80 (12.31)
BMI-SDS ^+e^	−3.04 (1.23)	−1.78 (0.79)	−1.09 (0.53)	−0.32 (0.62)	−2.72 (0.87)	−1.61 (0.52)	−1.11 (0.58)

+ based on KIGGS survey [41], T_0_ = admission, T_1_ = half of target weight, T_2_ = long term weight gain; close to target weight, HC = healthy controls. ^a^ Age T_0_ vs. HC: T(df = 58) = 3.12, *p* = 0.0028, ^b^ Height T_0_ vs. HC: T(df = 58) = 2.04, *p* = 0.046, ^c^ Weight T_0_ vs. HC: T(df = 58) = 11.17, *p* = 4.41 × 10^−16^, ^d^ BMI T_0_ vs. HC: T(df = 35.2 adjusted) = 12.95, *p* = 2.32 × 10^−15^, ^e^ BMI-SDS T_0_ vs. HC: T(df = 58) = 10.19, *p* = 1.56 × 10^−14^_._

**Table 2:** Comparison of analyte concentrations (metabolites, sum of hexoses and ratios) between (a) healthy controls (HC) and cases at each measurement time point and (b) between cases at each measurement time point. Metabolite concentrations of HC and cases in three measurement time points were compared using Kruskal-Wallis test (Table S1). Post-hoc pairwise comparisons were conducted with Mann-Whitney test and Friedman test.

Analytes	(a) Mann-Whitney Test *p*-Values			(b) Friedman Test *p*-Values			
	HC vs. T_0_	HC vs. T_1_	HC vs. T_2_	T_0_-T_1_-T_2_	T_0_ vs. T_1_ *	T_0_ vs. T_2_ *	T_1_ vs. T_2_ *
Acylcarnitines							
C10	0.989	**4.16 × 10^−5^**	0.153	0.004	**0.006**	1.000	**0.030**
C10:1	0.691	**1.81 × 10^−5^**	0.197	0.001	**0.001**	1.000	**0.006**
C12:1	0.834	**4.89 × 10^−5^**	0.337	0.005	**0.018**	1.000	**0.011**
C14:1	0.822	**8.68 × 10^−6^**	0.0536	0.001	**0.001**	0.910	**0.030**
C14:2	0.708	**7.55 × 10^−6^**	0.0919	0.003	**0.003**	1.000	**0.049**
C5-DC (C6-OH)	0.105	0.00117	0.856	0.001	**0.001**	0.077	0.435
C7-DC	0.248	**5.86 × 10^−7^**	0.224	3.62 × 10^−4^	**0.008**	1.000	**0.001**
C8	0.857	**9.09 × 10^−6^**	0.153	0.008	**0.011**	1.000	0.077
Amino acids							
Glutamic acid (Glu)	**2.49 × 10^−7^**	**1.08 × 10^−4^**	0.001	0.039	0.310	**0.039**	1.000
Lysophosphatidylcholines							
lysoPC a C14:0	0.031	**3.03 × 10^−8^**	0.034	0.001	**0.000**	0.510	**0.049**
lysoPC a C16:1	0.251	**1.87 × 10^−6^**	0.007	2.81 × 10^−4^	**0.000**	0.690	**0.018**
lysoPC a C17:0	**2.51 × 10^−5^**	**1.01 × 10^−8^**	0.002	0.010	0.310	0.510	**0.008**
lysoPC a C20:3	0.092	**8.52 × 10^−5^**	0.040	**1.20 × 10^−5^**	**0.000**	0.077	**0.039**
lysoPC a C28:0	0.030	0.002	0.311	0.005	**0.003**	0.510	0.178
Phosphatidylcholines							
PC aa C28:1	0.893	**2.09 × 10^−7^**	0.008	**2.10 × 10^−4^**	**0.000**	0.119	0.119
PC aa C30:0	0.168	**2.84 × 10^−6^**	0.048	0.003	**0.002**	0.259	0.259
PC aa C32:1	0.056	9.12 × 10^−4^	0.103	0.001	**0.000**	**0.049**	0.510
PC aa C32:2	4.63 × 10^−4^	0.003	0.241	**6.90 × 10^−5^**	**0.000**	**0.011**	0.510
PC aa C32:3	0.040	0.004	0.359	**9.80 × 10^−5^**	**0.000**	**0.049**	0.178
PC aa C34:3	0.110	0.006	0.354	**9.80 × 10^−5^**	**0.000**	**0.049**	0.178
PC aa C34:4	**1.48 × 10^−4^**	0.166	0.915	**1.03 × 10^−4^**	**0.000**	**0.006**	0.910
PC aa C38:0	0.192	**2.44 × 10^−8^**	0.003	**6.90 × 10^−5^**	**0.000**	0.510	**0.011**
PC aa C38:3	**1.99 × 10^−5^**	0.152	0.551	**2.00 × 10^−6^**	**0.000**	**0.000**	1.000
PC aa C38:6	0.156	**2.71 × 10^−6^**	0.054	**6.90 × 10^−5^**	**0.000**	0.510	**0.011**
PC aa C42:0	0.631	**2.65 × 10^−5^**	0.394	0.002	**0.002**	0.435	0.146
PC aa C42:1	0.958	**1.97 × 10^−5^**	0.418	4.87 × 10^−4^	**0.000**	0.795	**0.024**
PC ae C30:0	0.449	**3.68 × 10^−9^**	0.008	0.003	**0.006**	0.910	0.119
PC ae C34:0	0.822	**1.25 × 10^−7^**	0.0252	0.007	**0.006**	0.910	0.119
PC ae C34:1	0.267	**1.16 × 10^−6^**	0.277	0.001	**0.002**	1.000	**0.018**
PC ae C36:1	0.380	**1.17 × 10^−6^**	0.130	1.39 × 10^−4^	**0.000**	1.000	**0.003**
PC ae C36:2	0.078	**2.15 × 10^−6^**	0.0207	0.002	**0.006**	1.000	**0.006**
PC ae C38:2	0.591	**0.49 × 10^−7^**	0.004	4.00 × 10^−4^	**0.000**	1.000	**0.011**
PC ae C38:3	0.004	**5.09 × 10^−5^**	0.296	**4.00 × 10^−6^**	**0.000**	**0.018**	0.077
PC ae C38:5	0.079	**6.54 × 10^−7^**	**8.74 × 10^−5^**	0.001	**0.003**	**0.011**	1.000
PC ae C38:6	0.202	**6.98 × 10^−6^**	0.0313	0.080			
PC ae C40:3	0.205	**1.77 × 10^−4^**	0.228	0.001	**0.000**	0.510	**0.049**
PC ae C42:5	0.205	**1.87 × 10^−6^**	0.015	**1.76 × 10^−4^**	**0.000**	**0.030**	0.368
	**(a) Mann-Whitney Test** ***p*-Values**			**(b) Friedman Test** ***p*-Values**			
	HC vs. T_0_	HC vs. T_1_	HC vs. T_2_	T_0_-T_1_-T_2_	T_0_ vs. T_1_ *	T_0_ vs. T_2_ *	T_1_ vs. T_2_ *
Sphingomyelines							
SMOHC22:1	0.025	0.023	0.522	**6.90 × 10^−5^**	**0.000**	**0.011**	0.510
SMC24:1	0.277	3.69 × 10^−4^	0.012	0.003	**0.011**	**0.018**	1.000
Sums							
Carbohydrates							
H1	**2.43 × 10^−5^**	7.97 × 10^−4^	0.237	0.010	1.000	**0.011**	0.077
Ratios							
PUFA PC/SFA PC	**1.04 × 10^−5^**	**2.96 × 10^−6^**	0.002	0.465			
Total lysoPC/Total PC	**6.85 × 10^−5^**	0.001	0.133	0.007	0.119	**0.006**	0.910
Total SM/Total SM PC	6.12 × 10^−4^	0.376	0.515	0.001	**0.001**	0.062	0.595
Total SM/Total PC	6.46 × 10^−4^	0.322	0.509	0.001	**0.001**	0.077	0.435
Total SMOH/Total SM non OH	0.467	**2.56 × 10^−8^**	5.09 × 10^−4^	**5.00 × 10^−6^**	**0.000**	**0.002**	0.510

Abbreviations see Human Metabolome Database (HMDB): http://www.hmdb.ca/ [42]: (lyso)PC = (lyso)Phosphatidylcholines, C = Acylcarnitines, H1 = Hexoses, PUFA = Polyunsaturated Fatty Acids, SM = Sphingomyelines, SFA = Saturated Fatty Acids; Glu = Glutamic acid; HC (healthy controls): N = 25, T_0_: N = 35, T_1_: N = 26, T_2_: N = 22; * Bonferroni corrected; T_0_ = admission, T_1_ = half of target weight, T_2_ = long-term weight gain close to target weight.

**Table 4:** Results of time-course analysis (MEBA and two-way repeated ANOVA; significance threshold *p* = 2.16 × 10^−4^): Table 2. are listed. Two examples revealing heterogeneity: glutamic acid and tryptophan (see Figure 3).

Metabolite	MEBA Hotellings T^2^	ANOVA *F*-Value	ANOVA *p*-Value
PC aa C38:3	67.85	48.276	2.17 × 10^−10^
PC ae C38:3	55.56	31.016	3.24 × 10^−08^
lysoPC a C20:3	52.51	26.866	1.42 × 10^−07^
PC aa C34_4	48.73	23.604	5.04 × 10^−07^
PC aa C32_1	44.95	16.260	1.34 × 10^−05^
PC aa C30:0	42.83	15.564	1.90 × 10^−05^
PC aa C32:2	42.58	24.885	3.03 × 10^−07^
PC aa C28:1	40.21	23.351	5.58 × 10^−07^
PC ae C30:0	38.78	13.202	6.60 × 10^−05^
PC aa C34:3	38.01	19.997	2.32 × 10^−06^
C7-DC	37.37	13.471	5.70 × 10^−05^
PC aa C36:3	36.61	27.150	1.28 × 10^−07^
PC aa C38:6	36.22	17.798	6.36 × 10^−06^
PC aa C40:5	35.41	21.125	1.42 × 10^−06^
PC ae C34:0	34.41	11.609	1.62 × 10^−04^
PC aa C36:5	33.53	11.192	2.06 × 10^−04^
PC aa C32:3	32.76	17.760	6.48 × 10^−06^
PC ae C36:1	31.86	14.801	2.81 × 10^−05^
PC aa C36:6	29.82	18.329	4.96 × 10^−06^
LysoPC a C16:1	27.61	15.147	2.35 × 10^−05^

Abbreviations see Human Metabolome Database (HMDB) IDs: http://www.hmdb.ca/ [42].

## Data Availability

The data presented in this study are available in https://www.mdpi.com/2218-1989/11/1/7/s1.

## Supplementary Materials

The following are available online at https://www.mdpi.com/2218-1989/11/1/7/s1, Supplementary Table S1: Mean values of analyte concentrations (metabolites, sum of hexoses and ratios). Significance value of Kruskal Wallis test for controls, T0, T1 and T2 with MetaP. Supplementary Table S2: Group comparisons with non-parametric tests. Supplementary Table S3: Examined patterns of concentrations of metabolites (or their ratios) in controls and in T0, T1 and T2. Supplementary Table S4: Results from Multivariate Empirical Bayes Analyses (MEBA).
